# P-1877. Impact of Consultation Type on 30-Day Survival in Patients with Candidemia: Formal versus Automated Infectious Disease Consultation

**DOI:** 10.1093/ofid/ofaf695.2046

**Published:** 2026-01-11

**Authors:** Si-Ho Kim, Cheon Hoo Jeon, Yu Mi Wi, Kyong Ran Peck

**Affiliations:** Division of Infectious Diseases, Samsung Changwon Hospital, Sungkyunkwan University, Changwon, Kyongsang-namdo, Republic of Korea; Samsung Changwon Hospital, Changwon, Kyongsang-namdo, Republic of Korea; Samsung Changwon Hospital, Changwon, Kyongsang-namdo, Republic of Korea; Samsung Medical Center, Seoul, Seoul-t'ukpyolsi, Republic of Korea

## Abstract

**Background:**

Infectious disease (ID) consultation in candidemia has been associated with reduced mortality. This study evaluated 30-day survival based on the type of ID consultation: formal consultation and automated consultation linked to restricted antimicrobial prescriptions.

**Figure:**
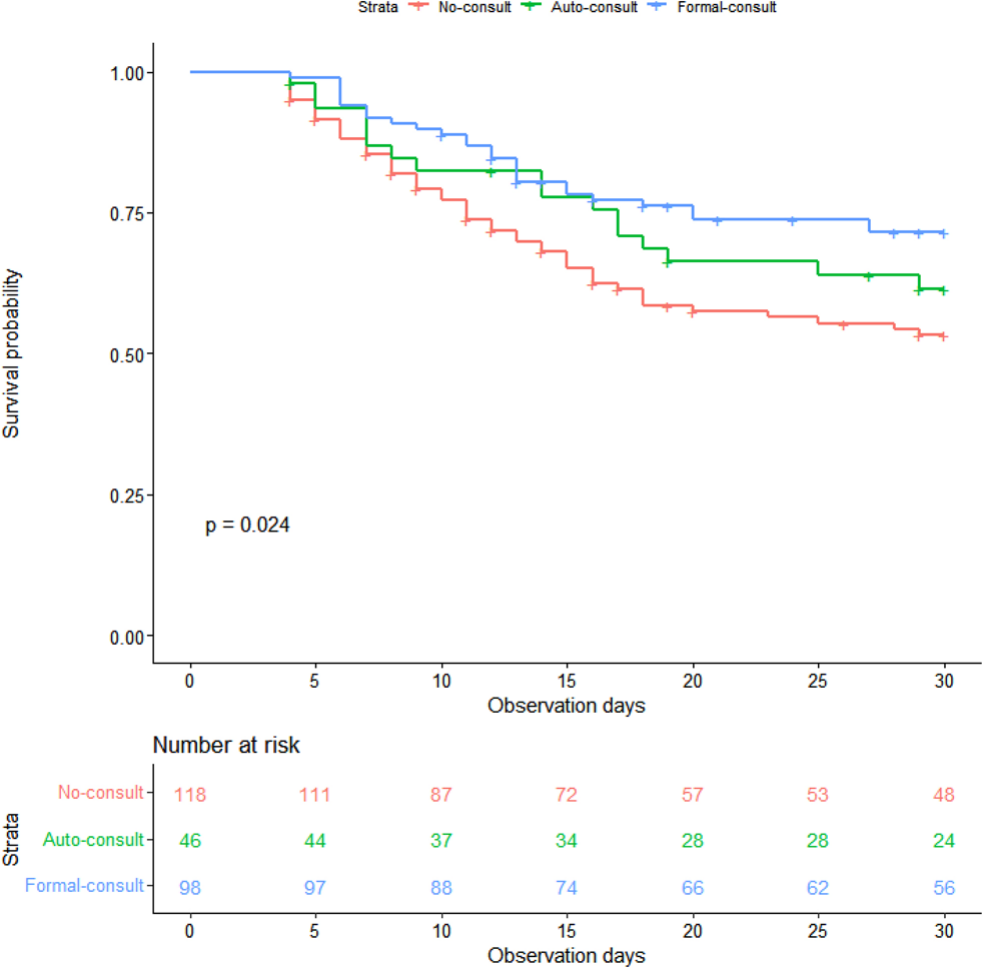
Overall 30-day survival rates among patients without consultation, with automatic consultation, and with formal consultation

**Methods:**

Adult patients with candidemia at Samsung Changwon Hospital (Jan 2015–Mar 2024) were included. Those who died within three days or had polymicrobial blood stream infection were excluded. Patients were grouped as: no consultation (No-consult), automated consultation (Auto-consult), and formal consultation (Formal-consult). Consultations within one week of candidemia onset were included. Automated consultation was triggered by prescribing restricted agents (echinocandins, glycopeptides, or carbapenems) and conducted via electronic records without bedside assessment. The primary outcome was 30-day survival. Secondary outcomes included appropriate antifungal use, follow-up cultures, source control, echocardiography, and ophthalmologic examination.

**Results:**

Among 262 patients, 118, 46, and 98 were in the No-, Auto-, and Formal-consult groups, respectively. Thirty-day survival was 53.2%, 61.4%, and 71.4% (P=0.024). This difference remained after adjustment, with significance only in the Formal-consult group (Auto-consult: HR 0.84, 95% CI 0.48–1.46; Formal-consult: HR 0.53, 95% CI 0.33–0.85). Significant differences were found in appropriate antifungal use (79.7%, 95.7%, 93.9%; P=0.001), follow-up cultures (66.9%, 91.3%, 95.9%; P< 0.001), and ophthalmologic exams (23.7%, 47.8%, 66.3%; P< 0.001). Fluconazole use was higher in the No-consult group (78.5%, 57.1%, 51.1%; P=0.001).

**Conclusion:**

Formal ID consultation was associated with improved 30-day survival in candidemia. Although a survival benefit was suggested in the automated consultation group, it did not reach statistical significance, indicating the need for additional strategies to enhance its effectiveness, particularly in resource-limited settings.

**Disclosures:**

All Authors: No reported disclosures

